# Characterization of an Impedance-Type Humidity Sensor Based on Porous SnO_2_/TiO_2_ Composite Ceramics Modified with Molybdenum and Zinc

**DOI:** 10.3390/s23198261

**Published:** 2023-10-06

**Authors:** Dalibor L. Sekulić, Tamara B. Ivetić

**Affiliations:** 1 Department of Power, Electronic and Telecommunication Engineering, Faculty of Technical Sciences, University of Novi Sad, Trg Dositeja Obradovića 6, 21000 Novi Sad, Serbia; dalsek@uns.ac.rs; 2 Department of Physics, Faculty of Sciences, University of Novi Sad, Trg Dositeja Obradovića 3, 21000 Novi Sad, Serbia

**Keywords:** humidity sensor, composite ceramics, solid-state synthesis, characterization, impedance

## Abstract

In this study, we report on the room-temperature characteristics of an impedance-type humidity sensor based on porous tin oxide/titanium oxide (SnO_2_/TiO_2_) composite ceramics modified with Mo and Zn. The SnO_2_/TiO_2_-based composites synthesized in the solid-state processing technique have been structurally characterized using X-ray diffraction, scanning electron microscopy, energy dispersive, and Raman spectroscopy. Structural analysis indicated the desired porous nature of the synthesized ceramics for sensing applications, with an average crystallite size in the nano range and a density of about 80%. The humidity-sensing properties were evaluated within a wide relative humidity range from 15% to 85% at room temperature, and the results showed that a better humidity response had a sample with Mo. This humidity-sensing material exhibits a linear impedance change of about two orders of magnitude at the optimal operating frequency of 10 kHz. Furthermore, fast response (18 s) and recovery (27 s), relatively small hysteresis (2.8%), repeatability, and good long-term stability were also obtained. Finally, the possible humidity-sensing mechanism was discussed in detail using the results of complex impedance analysis.

## 1. Introduction

Continuous progress in reducing fabrication costs and improving the reliability of consumer electronic circuits leads, inter alia, to an increased demand for different sensors, which convert physical or chemical quantities in various environments into electrical signals. On the other hand, the control and measurement of humidity, as one of the essential and permanent factors of the environment, are particularly important not only for human comfort but also for many different branches of industry and technology, as well as in medical applications. Therefore, there is a constant need to develop new humidity sensors based on cheaper and more stable functional materials with improved sensing performances over available ones.

In recent years, many kinds of sensing materials such as ceramics [1], polymers [2], carbon-based materials [3], and composites [4] have been studied for potential application in the fabrication of humidity sensors. On the other hand, thanks to the advantage of a large specific surface area, numerous nanostructured materials such as nanoparticles, nanotubes, nanorods, nanofibers, nanosheets, and nanoflowers are synthesized to enhance the humidity-sensing properties [5]. Among these different types of materials, ceramics based on various semiconductor metal oxides offer several advantages such as high chemical, mechanical, and thermal stability, while their porous nature enables rapid response dynamics and a broad range of operation [1,4]. The most investigated humidity sensors are binary metal oxide semiconductors, such as tin-, zinc-, anatase-, tungsten-, and hematite-based oxides, but also ternary oxides, like perovskites and cubic spinels of ferrites and stannates [6,7,8,9,10,11,12,13]. In addition, some oxygen-free compounds, zeolites, and clay minerals are also often used as humidity sensor materials [14]. In general, the humidity sensitivity of ceramic materials is mainly dependent on their microstructure. Namely, the quantity of adsorbed and condensed water depends on the available pore sizes and their distribution [15].

In most sensor applications, relative humidity (RH) measurement in air or other gases is most commonly used because it is generally simpler and thus cheaper than absolute humidity. The RH measurement is stated as a percentage and determined by the ratio of the amount of moisture content of air to the maximum, i.e., the saturated moisture level that the air can hold at a given temperature [16]. Most commercially available humidity sensors based on porous ceramics detect RH based on changes in the impedance of the used material as a sensing layer. The main mechanisms for humidity detection are the physical adsorption of water molecules at the surfaces or water condensation in mesopores of the ceramic sensing material, leading to a decrease in its impedance [17]. It is also possible to realize ceramic humidity sensors on the principle of measuring the change in capacitance of the sensing material with RH, but impedance (resistive)-type humidity sensors are preferable over capacitive-type ones.

Impedance-type humidity sensors offer several advantages, such as a wide operating range, low power consumption, and cost-effectiveness, as well as high sensitivity and fast response time. Namely, impedance-type humidity sensors can operate over a wide range of humidity levels and temperatures, making them suitable for various applications in different environments. Also, these humidity sensors typically consume less power than other types of humidity sensors, making them ideal for battery-operated (portable or wireless) devices and energy-efficient applications. Finally, impedance-type humidity sensors are relatively inexpensive to fabricate and maintain, making them an attractive option for budget-conscious applications. In general, the impedance-type humidity sensors based on ceramics are promising sensors for numerous applications due to their low cost, small size, and highly interchangeable properties [18].

Ceramic materials based on tin oxide (SnO_2_) and titanium oxide (TiO_2_), n-type wide band gap semiconductors, were the subject of numerous experimental studies over many years due to their potential applications in various electronic devices [19,20] and especially in humidity and gas sensors [14]. In sensor technology, the most interesting property of SnO_2_ is poor sinterability and low densification during production [21], which is advantageous in the case of sensor applications because porous microstructures contribute to greater chemisorptions of analytes (gas and water molecules) and increase the sensitivity of the ceramic-based sensors. Improving the sensitivity, stability, and selectivity of these sensors was mainly achieved by doping semiconductor metal oxides with noble metals (Pt, Pd, and Au) or other cheaper ones (Ni, Fe, and Cu) [22].

A very current, innovative, and at the same time much more cost-effective and simple way to improve ceramic-based sensor performance is the use of nanocomposites and heterostructures as sensing materials, which are obtained by combining two metal oxides, such as the recently tested SnO_2_/NiO [23] and TiO_2_/WO_3_ [24]. Semiconductor tin–titanium oxide SnO_2_/TiO_2_ (TTO) composites as solid solutions (Sn_x_Ti_1-x_)O_2_ are relatively new and interesting systems for sensor applications [25]. The surface morphology, structure, and elemental composition have a strong impact on the humidity-sensing performance of the TTO composite materials [26]. Although their thermodynamic and kinetic characteristics are generally known [27,28], there are still some basic properties that arise depending on the choice of synthesis method, e.g., cooling and heating cycles, the influence of pressure, additives, etc., which have not been fully explored. This largely relates to the unknowns of mass transport and sintering mechanisms, charge carriers and conductivity, etc.

The main goal of this study was to develop a good performance impedance-type humidity sensor based on a novel porous SnO_2_/TiO_2_ composite ceramic as a sensing material, synthesized using a simple and environmentally friendly technique from inexpensive precursors with lower sintering temperatures. Accordingly, this paper discusses the synthesis of TTO ceramic composites, their structure, morphology, and room temperature humidity-sensing characteristics, with special emphasis on the influence of molybdenum and zinc as functional additives. It is well-known that the addition of small amounts of ZnO to TiO_2_/SnO_2_ ceramics leads to the substitution of cations with Zn^2+^ to ensure sufficient electron mobility in the grains and also form defects that increase the resistance in the grain boundaries [29]. The use of the MoO_3_ modifier is new for this system and has been shown to benefit achieving higher porosity with good sensor characteristics. Therefore, synthesis optimization was performed to achieve higher porosity of ceramics and better adsorption of water molecules for their use as humidity-sensing elements with high chemical, mechanical, and thermal stability.

Here, ceramics based on tin oxide and titanium oxide (SnO_2_/TiO_2_) were modified with MoO_3_ and ZnO additives, which are also often used as flux agents [30], using a simple and economical mechanochemical procedure. The justification of our approach is confirmed by the results of the structure analysis and the impedance-type humidity-sensing properties. Microstructure characteristics of the prepared ceramic samples were investigated in detail using several techniques: X-ray diffraction, scanning electron microscopy, and Raman spectroscopy. By analyzing the impedance response at different RH levels and a low operating voltage (25 mV), the optimal frequency of the humidity sensor based on TTO ceramics was determined for achieving high linear sensitivity, a fast response recovery time, low hysteresis error, and good long-term stability. Finally, the humidity-sensing mechanism of TTO ceramics is discussed in detail using the results of the complex impedance spectroscopy, which may help boost the development of ceramic-based humidity sensors further.

## 2. Materials and Methods

Titanium–tin oxide (TTO) ceramics were synthesized by a mechanochemical procedure based on previous research [31]. Starting precursor powders of TiO_2_ anatase (Sigma-Aldrich; purity of 99.7% trace metals) and SnO_2_ rutile (Sigma-Aldrich, St. Louis, MO, USA; ~325 mesh powder; purity of 99.9% trace metals) were mixed in two separate procedures to obtain Sn_0.2_Ti_0.8_O_2_ stoichiometry. In the first procedure, 1 mol % MoO_3_ (Alfa Aesar; purity 99.9% metal basis) was added (hereinafter TTO:Mo sample), and in the second, 1 mol % MoO_3_ (Alfa Aesar, Ward Hill, MA, USA; purity 99.9% metal basis) and 1 mol % ZnO (Aldrich; purity 99.999% trace metal basis) were added (hereinafter sample TTO:Mo,Zn). The powder mixtures were wet milled in ethanol for 3.5 h using a ball mill (Retsch GmbH PM100, Haan, Germany), a 50 mL vial, balls of zirconium oxide 5 mm in diameter. The standard ball-to-powder mass ratio of BPR = 10:1, and a 100 rpm rotation speed was used. After air-drying for 24 h, the mechanically activated powder mixtures were calcined at 650 °C for 3 h to dispose of possibly residual ethanol used as a milling medium, which was followed by uniaxial pressing using a 10 mm diameter mold at 1.176 GPa. The formed pellets were finally sintered at 1100 °C for 4 h.

Bulk densities of sintered TTO samples were measured by pycnometric volume analysis and sample mass measurement. X-ray diffraction (XRD) was performed on a Rigaku SmartLab (Rigaku, Tokyo, Japan) system (Cu-K_α_ radiation, current 30 mA, voltage 40 kV) in the 2*θ* range from 10° to 80° (0.02° step size and 1°/min counting time). Scanning electron microscopy (SEM) and energy dispersive spectroscopy (EDS) were performed on a JEOL JSM-6460LV equipped with EDS (Oxford Instruments, Abingdon, UK). Raman scattering was obtained using a Renishaw Invia (Gloucestershire, UK) confocal Raman microscope and a 514 nm argon-ion laser at ambient temperature as the excitation source. The Raman signal was collected by a CCD camera in the frequency range of 100–1400 cm^−1^ with a spectral resolution of 2 cm^−1^ and an accumulation time of 3 s.

The impedance responses of the TTO samples were studied over a wide frequency range of 0.1 Hz to 10 MHz at different temperatures between 323 K and 523 K using a computer-controlled impedance analyzer (model Novocontrol Alpha-A, Montabaur, Germany) with a laboratory set of temperature control equipment (model Novotherm, Novocontrol Technologies, Montabaur, Germany). To perform these measurements, the surface of the sintered pellet was polished properly, and electrical contacts of silver paste were deposited on opposite sides of the prepared pellets and dried for 48 h. The obtained impedance spectra were fitted with a commercially available EIS Spectrum Analyzer [32] in order to establish a correlation between microstructure and electrical properties.

**Figure 1:**
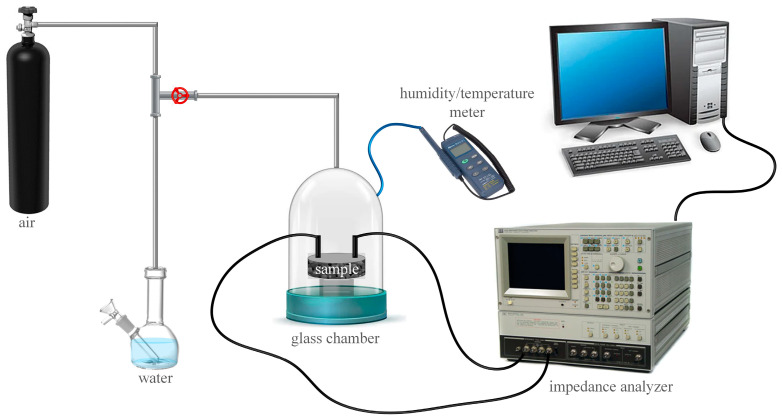
Schematic diagram of the measurement setup for testing the impedance response of humidity sensors based on ceramics.

To study the humidity-sensing performance, the prepared porous TTO ceramic pellet as sensing element was placed in a closed test glass chamber between two silver electrodes, which are connected to the computer-controlled impedance analyzer (model HP 4194A, Keysight, Santa Rosa, CA, USA) to measure the change in impedance with respect to RH at room temperature (25 ± 1 °C). The compressed air directed into the chamber was firstly dehydrated over silica gel and CaCl_2_, and then the humidity level was varied from 15% to 85% by bubbling air through water and mixing it with dry air [33]. In this measurement setup, whose schematic representation is given in Figure 1, RH and temperature were monitored by a commercial humidity and temperature probe (model Tecpel DTM–321, Taipei, Taiwan). Before the moisture was introduced to the sensor surface under study, the RH was calibrated by a commercial humidity sensor with a stable accuracy of about ±1% RH.

## 3. Results and Discussion

### 3.1. Crystal Structure and Morphology

**Figure 2:**
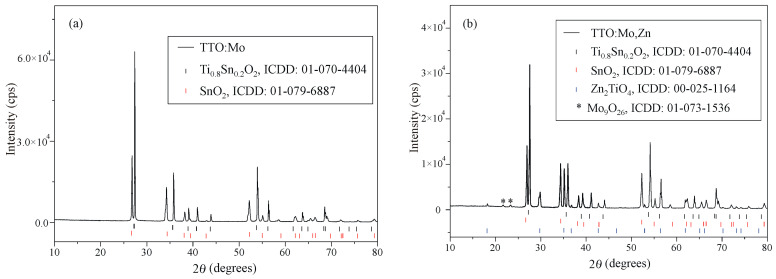
XRD patterns of the (**a**) TTO:Mo; (**b**) TTO:Mo,Zn samples.

Figure 2 shows the XRD diffraction patterns of the TTO:Mo and TTO:Mo,Zn samples. The diffraction peaks of the main phase are indexed to the tetragonal structure of the Sn_0.2_Ti_0.8_O_2_ solid solution (reference card number ICDD 01-070-4404, space group P42/mnm). The TTO:Mo impurity peaks belong to the SnO_2_ precursor phase (ICDD card No. 01-079-6889). Impurity peaks of TTO:Mo,Zn belong to precursor SnO_2_ (ICDD card No. 01-079-6889), newly formed Zn_2_TiO_4_ (ICDD card No. 00-025-1164), and Mo_9_O_26_ (ICDD card No. 01-073-1536) phases. The average crystallite size of the main Sn_0.2_Ti_0.8_O_2_ phase was calculated to be 60 nm and 46 nm of TTO:Mo and TTO:Mo,Zn, respectively, by observing its (110) diffraction peak using Scherrer’s equation [33]:

D = 0.89λ/βcos*θ*, (1)

where λ is the wavelength of CuK_α_ radiation (λ = 0.154 nm), β is the peak width at half the maximum intensity (FWHM) in the radians, and *θ* is the diffraction angle. The average crystallite size of the SnO_2_ phase was also calculated to be 44 nm and 35 nm of the TTO:Mo and TTO:Mo,Zn samples, respectively, while the Zn_2_TiO_4_ crystallites found in TTO:Mo,Zn were about 42 nm in size.

**Figure 3:**
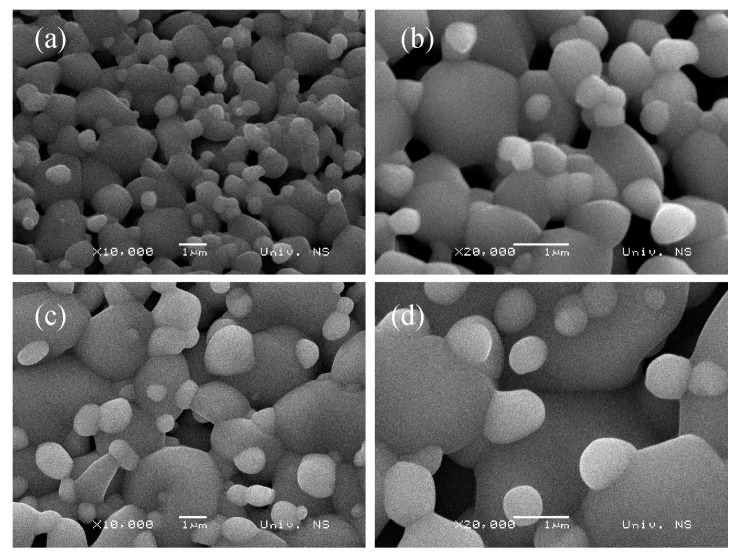
SEM images of the (**a**,**b**) TTO:Mo and (**c**,**d**) TTO:Mo,Zn samples with different magnifications.

**Figure 4:**
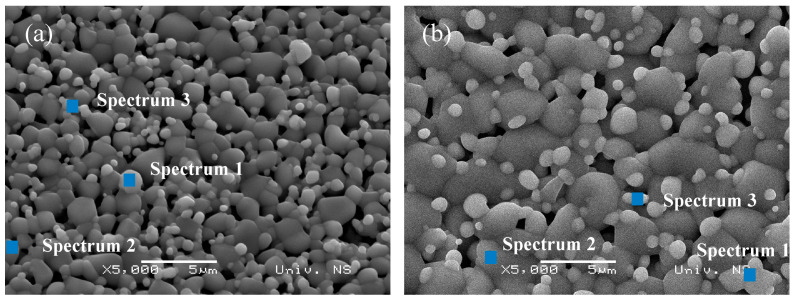
SEM images of the (**a**) TTO:Mo and (**b**) TTO:Mo,Zn samples with marked spots where EDS analysis was performed.

SEM images in Figure 3 and Figure 4 confirm the porous microstructure of the TTO samples composed of larger necked grains with much smaller spherical particles on top. The double cationic modification in TTO:Mo,Zn (Figure 3c,d) appears to stimulate grain growth and slightly reduce porosity [33]. The measured relative density (after sintering) of TTO:Mo and TTO:Mo,Zn was 81.92% and 82.82%, respectively, so the relative porosity was about 20%. Metal oxide ceramics with higher porosity should provide better adsorption of tested analytes, such as water molecules reaching the surface of the active semiconductor when testing changes in electrical properties with increasing relative humidity for sensor applications.

The influence of Mo and Zn cation introduction into the microstructure of the SnO_2_/TiO_2_ matrix compound is more clearly evident compared to the additive-free Ti_0.8_Sn_0.2_O_2_ (TTO) ceramics presented in our previous publication [31]. The double modification with MoO_3_ and ZnO seems to slightly reduce the porosity due to the greater tendency of ZnO toward densification and much easier diffusion [33]. The specific microstructure obtained with smaller spherical particles on top of larger ones is the result of compositional modification because such morphology was not seen in unmodified TTO [31].

The EDS measurement was performed at certain places on the surfaces of the synthesized TTO samples, as shown in Figure 4. The EDS characterization indicates areas with a higher and lower percentage of constituent atoms, as can be seen in Table 1. The EDS spectra did not show characteristic peaks corresponding to Mo and Zn atoms. Moreover, it was determined that they are forming secondary impurity phases that are incorporated into ceramic grains according to XRD findings. Therefore, the role of additives occurs mainly in the initial and middle stages of sintering [33].

**Figure 5:**
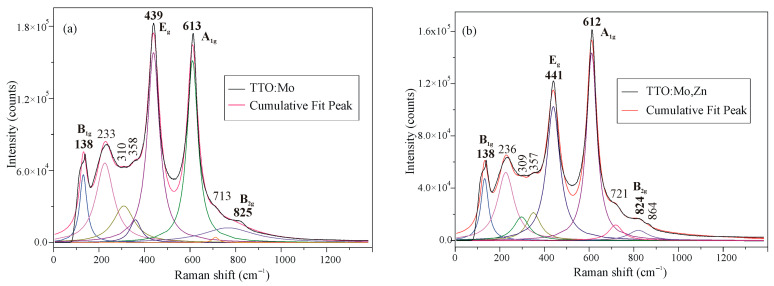
Raman spectra of the (**a**) TTO-Mo and (**b**) TTO-Mo,Zn samples.

The Raman spectra of the prepared TTO samples are shown in Figure 5. Up to nine peaks were resolved by Lorenzian line shape deconvolution and compared with references [31,34,35,36,37]. The peaks at around 138, 439, 613, and 825 cm^−1^ that dominate the spectra are attributed to *B*_1g_, *E*_g_, A_1g_, and *B*_2g_ vibration modes of TiO_2_, which can be explained by the higher content of TiO_2_ in the investigated solid solutions, which were made to have an approximate stoichiometry of the Ti_0.8_Sn_0.2_O_2_ compound. Each of the tetragonal TiO_2_ and SnO_2_ phases, according to the theory, shows the four aforementioned Raman active modes [31] at different frequencies. The double-cationic modification of the basic matrix (Ti_0.8_Sn_0.2_O_2_ solid solution) material was reflected in the intensity of Raman modes, which is generally reduced compared to the one cationic-modified sample and the appearance of an additional peak at 864 cm^−1^. Other smaller intensity peaks occur due to disorder-induced effects or second-order Raman scattering caused by the synthesis procedure, doping, etc. [31].

### 3.2. Impedance Spectroscopy

**Figure 6:**
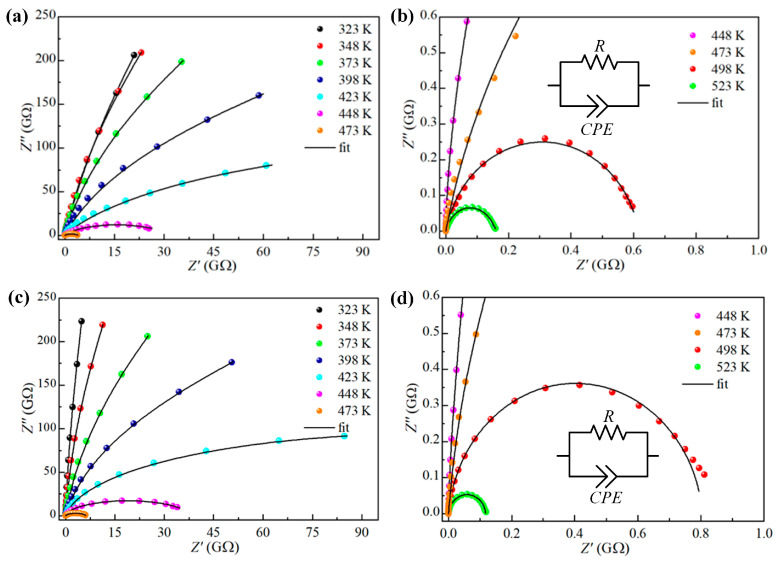
Experimental (scattered) and fitted (lines) impedance spectra for the (**a**,**b**) TTO:Mo, and (**c**,**d**) TTO:Mo,Zn samples.

In a wide frequency range from 0.1 Hz to 10 MHz and dry atmosphere, the complex impedance spectra (Z″ vs. Z′) were measured at different temperatures, and the obtained results are shown in Figure 6. At each temperature of the measurement, it is evident that the impedance spectrum of both TTO samples is characterized by the presence of only one semicircle arc with different radii. This kind of impedance response indicates that the grain boundary has a dominant effect on the conduction mechanism [38,39], which is probably due to the porous nature of the prepared samples, which was confirmed by structural analysis. In addition, all semicircle centers lie below the real axis of the impedance, and the origin of these depressed semicircles in the complex plane can be attributed to the presence of porosity and inhomogeneities in the TTO ceramic samples [40].

In Figure 6, it is also obvious that the radius of the semicircle arcs decreases rapidly as the temperature increases, which is a typical characteristic of most semiconducting oxides. In contrast to typical polycrystalline electroceramics, where impedance response is most often modeled with two parallel *R–C* circuits connected in series [41], in our case, the resulting impedance spectra were modeled with only one parallel *R–CPE* circuit. The constant phase element (*CPE*) was used to take into account the observed non-ideal Debye-like behavior of the prepared TTO ceramics as a result of the observed depressed semicircles in impedance response [31]. The validity of the proposed electrical circuit was confirmed by the close agreement of the experimentally obtained impedance spectra (scattered) and the curves obtained by fitting (lines) with an error of less than 3%, as seen in Figure 6. In the used model, element *R* represents the resistance, which was determined to be the order of GΩ for both samples under study at room temperature and dry atmosphere, indicating high electrical resistivity of the prepared TTO ceramic samples, which is a very desirable property for humidity-sensing applications [41].

### 3.3. Humidity-Sensing Properties

**Figure 7:**
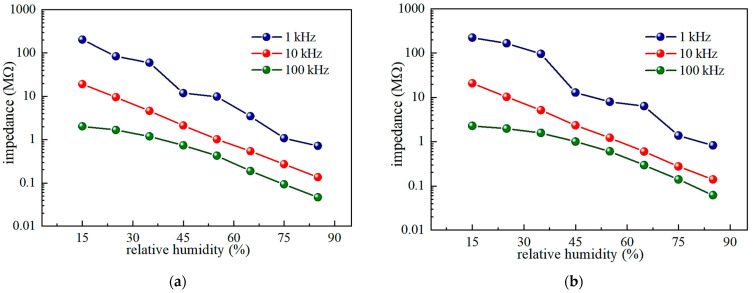
Dependence of impedance on RH for the (**a**) TTO:Mo, and (**b**) TTO:Mo,Zn ceramic samples at different frequencies at room temperature.

In order to study the humidity-sensing properties, the impedance of the prepared TTO sample was measured as a function of RH in the frequency range from 100 Hz to 1 MHz at room temperature (25°C), and the obtained results are shown in Figure 7. In the RH range between 15% and 85%, the impedance of both TTO composite ceramics decreases significantly with increasing frequency, but high sensitivity and good linearity were observed at a relatively low frequency of about 10 kHz. Therefore, for all the following measurements, 10 kHz was chosen as the optimal operating frequency of the humidity sensor based on porous TTO ceramics. At this frequency, the impedance of both samples changes by about two orders of magnitude, exhibiting pronounced sensitivity, but the TTO:Mo ceramic has a slightly better linearity and humidity response. Specifically, the best linear fit of the dependence of impedance on RH log*Z*(Ω) = 7.734 − 0.031×RH(%) with a correlation coefficient of R^2^ = 0.9987 was obtained for the TTO:Mo ceramic sample at a frequency of 10 kHz, while log*Z*(Ω) = 7.937 −0.036×RH(%) with R^2^ = 0.9892 was determined for the TTO:Mo,Zn ceramic sample at the same frequency. At higher frequencies, the impedance plots become flat because the direction of the applied electric field changes rapidly, and the polarization of the adsorbed water molecules cannot catch up [42,43].

**Figure 8:**
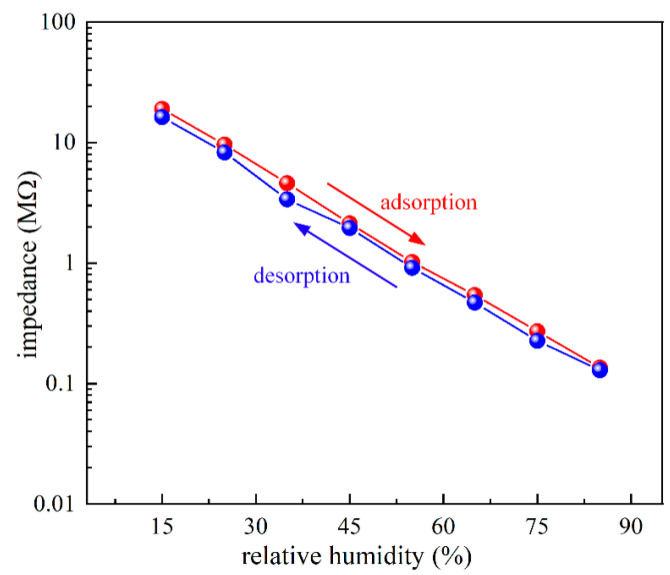
Room humidity hysteresis curve of the TTO:Mo ceramic material at a frequency of 10 kHz at room temperature.

Based on the above results, the humidity sensors based on TTO:Mo ceramic material can possess better sensing properties in the tested RH range, and thus this sample was used to evaluate adsorption/desorption ability and repeatability. At room temperature and determined operating frequency of 10 kHz, the humidity hysteresis characteristic given in Figure 8 shows a small difference between impedance during the adsorption and desorption processes, indicating good reliability of the porous TTO:Mo ceramic as a sensing material. Furthermore, the impedance of the desorption process is slightly lower than the adsorption process due to the fact that a relatively longer time is required to desorb the water molecules [44]. The maximum humidity hysteresis error was calculated to be about 2.8%. In general, a smaller value of the hysteresis error in a humidity sensor leads to increased reliability and measurement consistency, which are two important parameters for sensor performance [45]. Large hysteresis is usually observed due to improper interaction between water molecules and the porous humidity-sensing layer [46]. Therefore, the hysteresis issue limits the practical applications of humidity sensors, especially for precision measurement applications. Namely, hysteresis represents the memory effect aspect of the humidity sensors, where the accuracy has an offset that depends on the previous RH value and the offset varies depending on what the prior RH was.

**Figure 9:**
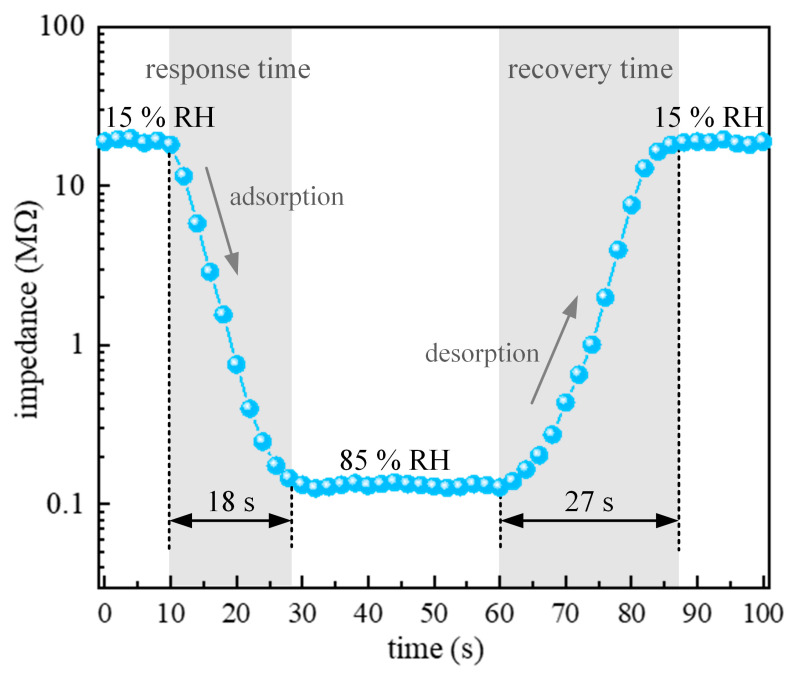
A single cycle response recovery characteristic of the TTO:Mo ceramic sample at a frequency of 10 kHz at room temperature.

In order to evaluate the sensor performance for real-time humidity monitoring, the room temperature response recovery characteristic for one cycle was determined and depicted in Figure 9. In general, the response and recovery times indicate how long the measurement takes to reach 90% of the final impedance value when there is an instant change in the RH being measured. At the previously determined operating frequency of 10 kHz, the response time (humidification from 15% to 85% RH), defined as the time needed to achieve 90% of the total impedance change after humidity introduction, was determined to be 18 s, and the recovery time (desiccation from 85% to 15% RH), defined as the time taken by the sensor to return to 90% of the initial impedance value after returning to dry airflow, was measured to be 27 s for synthesized porous TTO:Mo ceramics as humidity-sensing materials. Comparing these values with the previously published ones, see Table 2, the obtained experimental results indicate good performance of ceramic humidity sensors based on porous TTO:Mo and generally validate our approach. This relatively fast response and recovery behavior can be attributed to the presence of a high surface area and pore volume, which facilitate the adsorption and desorption of water molecules on the internal and external surface of the material [47,48].

**Figure 10:**
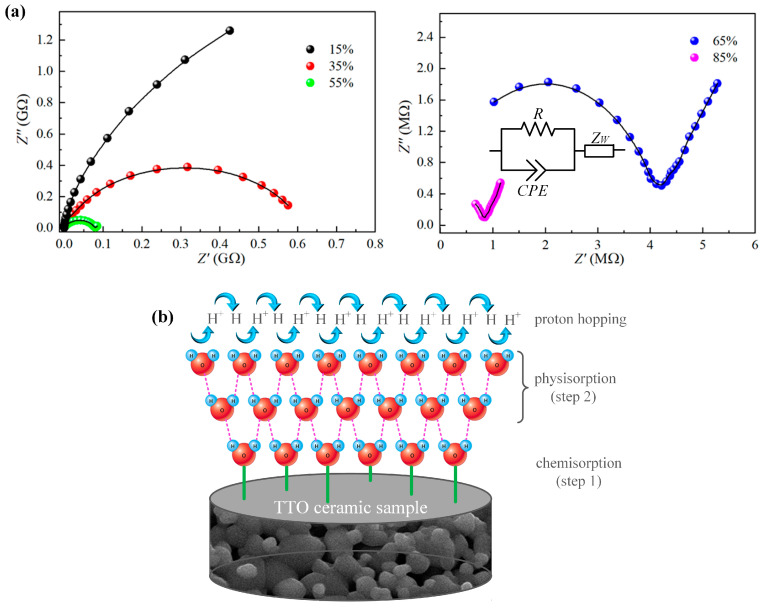
(**a**) The room temperature experimental (scattered) and fitted (lines) impedance spectra for the TTO:Mo sample under different RH levels. (**b**) Schematic representation of the humidity-sensing mechanism for the porous TTO ceramic samples.

Complex impedance spectra are often used to study the sensing process of impedance-type humidity sensors. Figure 10a shows the room temperature impedance spectra of the TTO:Mo sensing materials, which were obtained in the frequency range from 100 Hz to 1 MHz under different RH levels. The observed variation in impedance spectra suggests different water absorption mechanisms related to electrical conductivity and polarization that occur in the TTO ceramics [54]. In general, the humidity-sensing mechanism, whose schematic diagram is given in Figure 10b, can be described by two successive processes: chemisorption and physisorption [55]. When RH is low (15–35% RH), it is obvious that the spectrum is characterized by an incomplete semicircle that becomes complete with increasing humidity. This phenomenon indicates a non-Debye behavior, which can be modeled by an equivalent circuit of parallel resistor *R* and constant phase element *CPE*. The radius of the semicircle decreases with increasing RH and results in a decrease in intrinsic impedance, which is mainly due to the interaction between the sensing material and water molecules. At low RH, the adsorption of water molecules on the surface of the crystalline grains of the sample takes place via a dissociative chemisorption process, which results in the formation of hydroxyl groups (OH^−^) at the surface of the sensing layer (step 1 in Figure 10). As a result, the electrons are accumulated at the sample surface and consequently, the impedance of TTO ceramics decreases with an increase in RH [56]. However, a continuous water layer is not yet formed on the sensing material surface, and the conduction of free protons (H^+^) from one position to another on the surface is difficult, resulting in the still relatively high impedance [53].

With a further increase in RH (55–85% RH), each impedance spectrum is composed of a part of a semicircle in the high-frequency region and a straight line in the low-frequency region. More specifically, the arc length of the semicircle becomes shorter, and the straight line becomes longer with increasing humidity, in which the straight line is attributed to Warburg impedance *Z*_w_ caused by ionic (H_3_O^+^) conduction at the electrode/sensing material interface [5,57]. H_3_O^+^ ions could be formed by H^+^ protons and water molecules [44]. When RH increases significantly, subsequent layers of water molecules will be physically adsorbed on a chemisorbed layer (step 2 in Figure 10). These physically adsorbed water layers show a liquid-like behavior, and protons (H^+^) move freely. In this case, the conduction process occurs mainly by the Grotthuss transport mechanism [56]. The free movement of H^+^ along with the water layer causes a further significant decrease in the impedance of the studied TTO ceramics.

**Figure 11:**
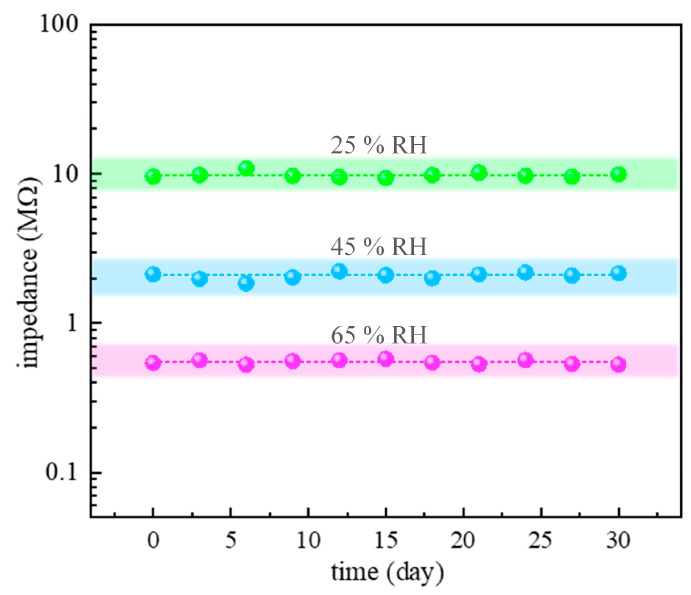
The long-term stability curves of porous TTO:Mo ceramics as sensing layers monitored at different humidity conditions for 30 days.

Finally, long-term stability was examined as one of the most imperative parameters for any sensing application. Namely, the long-term stability of humidity sensors is their essential capability for carrying out long-term data collection for environmental monitoring. The main factors that affect the stability of the sensing material are the uncontrolled grain growth during the service period of the sensor and the irreversible reactions between the material surface and some molecules in the environment [58]. For that purpose, the sensing response of the TTO:Mo sample was measured repeatedly once in three days under fixed humidity levels (25%, 45%, and 65% RH) at an optimal operating frequency of 10 kHz in a period of 30 days. As can be seen in Figure 11, there are slight fluctuations in the impedances with time (less than ± 4.2%), which directly indicates good stability and reliability. The determined fluctuation over 30 days of testing implies a relatively small variability of the humidity sensor output over a long period in similar environmental conditions or under multiple measurements. Therefore, the obtained stability characteristics confirm that porous TTO:Mo ceramics are promising materials for applications in low-cost impedance-type humidity sensors.

## 4. Conclusions

In summary, this paper reports on the microstructure and humidity-sensing properties of novel TTO-based ceramics modified by functional additives (Mo, Zn), which were synthesized by the solid-state mechanochemical method from relatively inexpensive starting precursors. XRD, SEM, EDS, diffuse reflectance, Raman, and impedance spectroscopy were used for characterization. The XRD pattern showed the main phase solid solution composition based on the rutile structure, which was confirmed by the Raman analysis. SEM and EDS analysis revealed the formation of a highly desirable porous microstructure with the incorporation of Mo and Zn cations through impurity phases. The evaluation of humidity-sensing performance demonstrated the nearly linear decrease in impedance of both TTO samples with increasing RH in the whole range from 15% to 85% at a frequency of 10 kHz, which is chosen for operating frequency. As the TTO-based ceramics modified by Mo exhibited a slightly better linearity and humidity response, this sample was, therefore, selected for further detailed investigation of its humidity properties. Experimental results revealed that the impedance-type humidity sensor based on TTO:Mo ceramics possesses fast response/recovery times (about 18 s/27 s) with small hysteresis errors (about 2.8%) and good long-term stability. Therefore, porous TTO ceramics appear to be very promising materials for the design and fabrication of low-cost impedance-type humidity sensors with good performance in comparison with conventional sensors.

## Figures and Tables

**Table 1:** Results of the EDS elemental composition findings (weight %) of the TTO samples (taken from the spots indicated in Figure 4).

Sample	TTO:Mo			TTO:Mo,Zn		
Spectrum	O	Ti	Sn	O	Ti	Sn
Spectrum 1	24.95	21.46	53.59	47.04	34.12	18.84
Spectrum 2	35.66	40.28	24.06	41.50	41.72	16.79
Spectrum 3	36.36	30.34	33.30	31.95	24.26	43.79

**Table 2:** Performance comparison of the TTO-based ceramic humidity sensor with other humidity sensors available in the literature.

Sensing Material	Detection Range	Sensitivity	Response Time	Recovery Time	Hysteresis Error	Reference
TiO_2_-SnO_2_	20–90% RH	~10^3^	20 s	-	-	[49]
SnO_2_-TiO_2_	10–95% RH	12.374	19.1s	181 s	-	[26]
ZnO/SnO_2_	11–95% RH	~10^4^	8 s	35 s	6.6%	[5]
NaTaO_3_/TiO_2_	11–95% RH	~10^4^	13 s	9 s	8.3%	[50]
TiO_2_-SnS_2_	11–93% RH	~10^3^	60 s	-	-	[51]
SmFeO_3_@MoS_2_	11–95% RH	~10^5^	1.5 s	29.8 s	2%	[52]
(In+Nb)-doped HfO_2_	11–94% RH	3612	20 s	50 s	6.79%	[53]
CNT	15–98% RH	172	12 s	47 s	3.6%	[45]
TTO:Mo	15–85% RH	~10^2^	18 s	27 s	2.8%	This work

## Data Availability

Not applicable.
